# Neuroactive steroids for the treatment of depression and anxiety: gaps and promises

**DOI:** 10.1007/s00406-026-02196-z

**Published:** 2026-01-24

**Authors:** Rainer Rupprecht, Cornelius Schüle, Thomas Gudermann

**Affiliations:** 1 Department of Psychiatry and Psychotherapy , University Regensburg , Universitätsstr. 84, 93053 Regensburg, Germany; 2 Department of Psychiatry and Psychotherapy, LMU University Hospital, Ludwig-Maximilians-University , Nußbaumstr. 7, 80336 Munich, Germany; 3 Walter Straub Institute of Pharmacology and Toxicology , Ludwig-Maximilians-University , Goethestr. 33, 80336 Munich, Germany; 4 M1 Munich Medicine Alliance , Bavariaring 19, 80336 Munich, Germany

In September 2025, Biogen received approval for marketing of zuranolone (Zurzuvae^®^) from the European Comission for the treatment of postpartum depression, following approval from the Medicines and Healthcare Products Regulatory Agency (MHRA) in the UK in August 2025 and the US Food and Drug Administration (FDA) in August 2023. Zuranolone is a synthetic 3α-reduced neuroactive steroid and an allosteric modulator of γ-aminobutyric acid type A (GABA_A_) receptors. As such, it is time to put the approval of zuranolone in the context of unmet treatment needs and the neurosteroid research field and its potential for the treatment of affective disorders.

Affective disorders such as (postpartum) depression and anxiety disorders impose a great burden on individual patients but also on society due to immense socioeconomic consequences. Besides various treatment options pharmacotherapy is still a major pillar for treatment of severe affective disorders. Whereas benzodiazepines, which are also positive allosteric modulators of GABA_A_ receptors, are frequently used for acute treatment of anxiety, their medium and long-term use is limited by tolerance development and abuse liability. Antidepressants such as selective serotonin (SSRIs) and selective serotonin/norepinehprine reuptake inhibitors (SNRIs) are used not only for treatment of depression but also for anxiety disorders. However, a major unmet need is their latency until a clinical meaningful effect, which usually lasts several weeks. This is where the neurosteroid field comes into play.

The principle of allosteric modulation of GABA_A_ receptors by 3α-reduced metabolites of progesterone, e.g., allopregnanolone, was discovered in 1986 [1]. Intriguingly, such steroids can be synthesized in the brain, which then are called neurosteroids [2]. This was the starting point for fruitful mostly preclinical research in the field, showing that such endogenous steroids can exert anxiolytic and/or antidepressant properties.

As such, the idea was born that neurosteroids might also be used as therapeutic agents. For such steroids not entirely made in the brain, the term „neuroactive steroids“ has been coined [2]. The development of suitable gas chromatography/mass spectrometry techniques enabled the accurate measurement of these steroids in both plasma and cerebrospinal fluid in depressed patients. These studies revealed an altered composition of neurosteroids in depression [3, 4]. Intriguingly, SSRIs were found to alter neurosteroid composition in these studies [3, 4] but also to directly interfere with the activity of the 3α-hydroxysteroid oxidoreductase, a neurosteroidogenic enzyme [5]. As such, enhancement of endogenous neurosteroidogenesis may constitute an alternative approach to the application of exogenous 3α-reduced steroids.

The translocator protein 18 kDa (TSPO) has been identified as a major determinant of steroidogenesis [6]. Moreover, a TSPO ligand has been shown to increase GABAergic transmission in rodents and to exert anxiolytic effects in humans in a first proof-of-concept study in the absence of benzodiazepine inherent side effects [7]. In France, etifoxine, which is mixed GABA_A_ receptor/TSPO ligand, is available for the treatment of anxiety syndromes [8].

From a pharmacological point of view it is plausible that 3α-reduced neurosteroids as positive allosteric modulators of GABA_A_ receptors act as anxiolytics and may ameliorate postpartum depression, given the huge drop in progesterone and related metabolites following delivery. However, it is less clear why they should be effective in major depression as benzodiazepines do not exert prominent antidepressant effects. However, 3α-reduced neurosteroids target also extrasynaptic GABA_A_ receptors with different subunit composition and region specific expression mediating tonic inhibition, whereas benzodiazepines predominantly act via synaptic receptors related to phasic inhibiton. This may also explain putative differences in side effect profiles.

Brexanolone, which is allopregnanolone prepared for intravenous infusion, was the first approved neurosteroid for the treatment of postpartum depression. Zuranolone, a modification suitable for oral administration, has been shown to rapidly decrease depression symptoms both in postpartum depression and in major depressive disoder [9, 10], which would fill the gap until the prolonged effects of antidepressants.

Besides efficacy, side effects have also to be considered. In the zuranolone study portfolio, the compound has only been administered for a 14 days time period. This raises the question of tolerance development and abuse liability upon a more prolonged administration. In the US, zuranolone has been classified as a Class IV controlled substance by the Drug Enforcement Agency (DEA). Most common side effects are somnolence, dizziness and sedation. Notably, FDA approval for zuranolone in major depressive disorder (MDD) has not been granted yet in view of these issues and questions concernig efficacy.

Regarding TSPO ligands, a first proof-of-concept trial in depression is currently under way and first results are expected in 2026. It remains to be determined whether this approach yields similar or different efficacy and/or side effects.

After 40 years of research the neurosteroid field has the potential for novel treatment options in affective disorders, which is really good news.
